# The efficacy and safety of olanzapine combined with repetitive transcranial magnetic stimulation (rTMS) in patients with comorbid anxiety–depression and sleep disorders

**DOI:** 10.1097/MD.0000000000050199

**Published:** 2026-10-30

**Authors:** Deyang Yin, Da Yang, Xuan Hou, Ying Liu

**Affiliations:** a Department of Psychosomatic Medicine, Dazhou Central Hospital, Dazhou, Sichuan, China.

**Keywords:** Anxiety, clinical efficacy, depression, olanzapine, repetitive transcranial magnetic stimulation, sleep disorders

## Abstract

This retrospective study non-randomized cohort study evaluated the efficacy and safety of adjunctive repetitive transcranial magnetic stimulation (rTMS) combined with olanzapine in adults with comorbid anxiety–depression and sleep disorders treated between April 2019 and March 2025. A total of 210 patients were included and allocated, according to the treatment actually received in routine clinical practice, to an olanzapine monotherapy group (n = 102) or a combined treatment group receiving olanzapine plus rTMS for 4 weeks (n = 108). Baseline demographic and clinical characteristics, including baseline sleep, anxiety, and depression severity, were comparable between groups. After 4 weeks, actigraphy-derived sleep parameters favored the combined group, including higher sleep efficiency (80.31% ± 9.85% vs 76.85% ± 10.53%), longer total sleep time (404.26 ± 14.52 vs 398.26 ± 13.52 minutes), and fewer nighttime awakenings (3.78 ± 0.94 vs 4.12 ± 0.85). Consistent with these objective findings, greater reductions were observed in patient-reported sleep scales, with lower posttreatment Pittsburgh Sleep Quality Index (8.49 ± 1.24 vs 9.05 ± 1.27) and ISI scores (11.47 ± 1.28 vs 11.91 ± 1.31). Both groups demonstrated improvement in mood and anxiety symptoms; however, reductions in Hamilton Depression Rating Scale (13.51 ± 1.85 vs 14.12 ± 1.49) and Hamilton Anxiety Rating Scale scores (12.61 ± 1.78 vs 13.37 ± 1.69) were greater with combined therapy, accompanied by higher overall clinical efficacy and improved World Health Organization Quality of Life – Brief Version physical and psychological domains. Metabolic indices (body mass index, fasting blood glucose, and lipids) were similar between groups at baseline and follow-up. Headache occurred more frequently with rTMS, whereas other adverse events were infrequent and comparable. These findings support the short-term effectiveness of olanzapine plus rTMS for improving sleep and affective symptoms without additional metabolic burden. However, the retrospective non-randomized design, potential residual confounding, and 4-week follow-up require cautious interpretation and confirmation in randomized long-term studies.

## 1. Introduction

Comorbid anxiety and depression were among the most prevalent and debilitating psychiatric conditions, affecting millions of individuals worldwide and contributing substantially to the global burden of disease.^[1]^ Patients suffering from these co-occurring disorders frequently experience heightened symptom severity, poorer functional outcomes, increased healthcare utilization, and elevated risk of suicidality compared to those with either condition alone.^[2]^ Importantly, disturbances in sleep were highly prevalent within this population, with insomnia and other sleep disorders documented as both predisposing factors and clinical sequelae of comorbid anxiety and depression.^[3]^ Left untreated, sleep dysfunction exacerbated mood and anxiety symptoms, impaired cognitive and psychosocial functioning, and undermined quality of life, compounding the challenge of effective management in clinical practice.^[4]^

Current clinical guidelines recommended a combination of pharmacotherapy and psychotherapy for the treatment of comorbid anxiety and depression, with selective serotonin reuptake inhibitors, serotonin–norepinephrine reuptake inhibitors, and atypical antipsychotics, such as olanzapine, being widely utilized as 1st- and 2nd-line pharmacologic strategies.^[5]^ Olanzapine, an atypical antipsychotic with antagonist activity at dopamine D2 and serotonin 5-hydroxytryptamine 2A and 2C receptors, had been shown to possess anxiolytic and antidepressant properties when used as monotherapy or as an augmentation agent.^[6]^ Nevertheless, olanzapine’s use was frequently limited by metabolic side effects and, in some cases, inadequate response or remission rates – particularly in the context of comorbid sleep disturbances, which might be less responsive to pharmacological intervention alone.^[7]^

To address limitations of conventional pharmacotherapies, neuromodulatory techniques had been increasingly explored as adjunctive interventions in mood and anxiety disorders.^[8]^ Among these, repetitive transcranial magnetic stimulation (rTMS) – a noninvasive brain stimulation modality that uses rapidly changing magnetic fields to modulate cortical excitability – had gained regulatory approval for the treatment of major depressive disorder and treatment-resistant depression, supported by a growing evidence base demonstrating efficacy and safety.^[9]^ Recent studies^[10,11]^ further suggested rTMS might benefit patients with generalized anxiety disorder, as well as those with marked sleep impairment, by targeting dysfunction in the dorsolateral prefrontal cortex (DLPFC) and interconnected affective-limbic circuitry. Thus, the combined use of rTMS and pharmacotherapy offered a promising multimodal strategy to address the multidimensional neurobiology of anxiety–depression comorbidity with sleep dysfunction. Despite these theoretical and empirical advances, there remained a paucity of rigorous studies evaluating the clinical value and safety profile of olanzapine combined with rTMS in patients presenting with both anxiety–depressive symptoms and sleep disorders. The present study aimed to fill this gap by retrospectively analyzing the efficacy and safety of adjunctive low-frequency rTMS in combination with olanzapine treatment among adults with comorbid anxiety–depression and sleep impairment in a naturalistic clinical setting. We hypothesized that the addition of rTMS would enhance improvements in sleep quality, mood, and anxiety symptoms, as well as overall quality of life, without significantly increasing adverse metabolic or neurological effects compared to olanzapine monotherapy. By clarifying the therapeutic potential of this combined intervention, our findings could inform evidence-based approaches for complex presentations that remained only partially responsive to standard management.

## 2. Methods

### 2.1. Study design and population

This study was approved by the Ethics Committee of Dazhou Central Hospital. A total of 210 patients diagnosed with anxiety and depression, in addition to sleep disorders, who received treatment at our hospital between April 2019 and March 2025 were included in the study. Based on different treatment approaches, patients were divided into the olanzapine group (n = 102) and the combined rTMS group (n = 108). Patient data were collected through the hospital’s electronic medical record system. The inclusion criteria were as follows: patients aged 18 to 70 years, meeting the diagnostic criteria for comorbid anxiety and depression according to the Diagnostic and Statistical Manual of Mental Disorders, fifth edition; with Hamilton Anxiety Scale (HAMA ≥ 14) and Hamilton Depression Scale (HAMD ≥ 17) scores meeting the threshold; Pittsburgh Sleep Quality Index (PSQI > 7); no prior rTMS treatment; and complete medical records. Exclusion criteria: secondary depression caused by organic diseases, medications, or other psychiatric disorders; patients with schizophrenia or bipolar disorder; history of severe suicidal tendencies; severe cardiovascular, hematopoietic, hepatic, or renal dysfunction; history of alcohol or substance dependence; pregnant or breastfeeding women; and contraindications to rTMS (e.g., intracranial metal implants or history of epilepsy). This study was approved by the Ethics Committee of Dazhou Central Hospital. The procedures were conducted in accordance with the ethical standards set forth by the Committee on Human Experimentation and the Helsinki Declaration of 1964, as revised in 2013. Informed consent was waived by the Ethics Committee of Dazhou Central Hospital for this retrospective study due to the exclusive use of de-identified patient data, which posed no potential harm or impact on patient care. This waiver was approved by the institutional review board and ethics committee of our institution in accordance with regulatory and ethical guidelines pertaining to retrospective studies.

Group assignment was based on the treatment actually received in routine clinical practice rather than investigator-directed randomization. Patients who received olanzapine alone were assigned to the olanzapine group (n = 102), whereas patients who received adjunctive rTMS in addition to olanzapine were assigned to the combined rTMS Group (n = 108). The decision to add rTMS was made by the treating physicians after routine clinical evaluation and consideration of patient preference, treatment accessibility, tolerance, and the absence of rTMS contraindications. Importantly, both groups met the same diagnostic thresholds and inclusion/exclusion criteria, and no additional disease-specific indication or baseline severity threshold was used to allocate patients to the combined treatment group. Baseline demographic characteristics and disease severity indicators, including HAMD, HAMA, PSQI, and Insomnia Severity Index (ISI) scores, were extracted before treatment and compared between groups to evaluate baseline comparability. Because of the retrospective, non-randomized design, these comparisons were used to describe group balance but could not fully eliminate the possibility of selection bias or residual confounding.

### 2.2. Treatment methods

In the olanzapine group, patients were treated orally with olanzapine tablets (National Drug approval no. H20203425, Sichuan Kelun Pharmaceutical Co., Ltd.), 1 tablet once daily, with dose adjustments based on tolerance. The treatment course lasted 4 weeks.

In the combined rTMS Group, in addition to the treatment administered in the olanzapine group, patients received supplementary low-frequency rTMS therapy. Prior to administering rTMS treatment, stimulation coordinates were determined according to the U.S. Food and Drug Administration-approved protocol. Using a figure-8 coil (AirFilm Coil Fast Edition, Magstim) with the Magstim Rapid^2^ stimulator (Magstim), TMS pulses were delivered to the left motor cortex and DLPFC. The coil was placed between the left frontocentral and left central electrode positions, which optimally elicited motor evoked potentials in the right abductor pollicis brevis muscle, thereby precisely localizing the motor cortex. The DLPFC target was identified using the 5-cm rule. Both the motor cortex and DLPFC stimulation sites were marked to ensure consistent coil positioning. The coil handle was oriented perpendicular to the central sulcus, tilted 45° posteriorly from the midline. The rTMS treatment strictly adhered to the U.S. Food and Drug Administration-approved protocol for depression: a 4-week course (5 sessions/wk), totaling 20 sessions. DLPFC stimulation parameters were set as follows: intensity at 120% of the motor threshold, frequency at 10 Hz, and a total session duration of 37.5 minutes.

### 2.3. Sleep state

After 4 weeks of treatment, the patients’ sleep quality was monitored using an actigraph (wGT3X-BT, ActiGraph), which provided relatively objective assessments of sleep efficiency, nighttime awakenings, and total sleep time. The PSQI was used to evaluate overall sleep quality, covering 7 dimensions: subjective sleep quality, sleep latency, sleep duration, sleep efficiency, sleep disturbances, use of sleep medication, and daytime dysfunction.^[12]^ The questionnaire consisted of 19 self-rated items, with 5 additional questions filled out by others (not included in the scoring). Each dimension was scored on a scale of 0 to 3, with a total score ranging from 0 to 21. A higher score indicated poorer sleep quality.

The ISI was used to assess the severity of insomnia symptoms.^[13]^ The questionnaire included 7 items addressing difficulty falling asleep, sleep maintenance, early awakening, satisfaction with sleep, daytime functional impairment, impact on quality of life, and distress caused by insomnia. Each item was scored on a scale of 0 to 4, with a total score ranging from 0 to 28. Higher scores indicated more severe insomnia. Based on the total score, insomnia could be classified into 4 levels: 0 to 7: no clinically significant insomnia; 8 to 14: mild insomnia; 15 to 21: moderate insomnia; and 22 to 28: severe insomnia.

### 2.4. Symptoms of anxiety and depression

The HAMD consisted of 17 items covering depressive mood, guilt, suicidal ideation, difficulty falling asleep, restless sleep, early awakening, reduced work and interests, and other aspects.^[14]^ The assessment was typically conducted by trained nurses through a semi-structured interview, combining patient self-reports and clinical observations. Each item was scored on a scale of 0 to 2 or 0 to 4, with higher total scores indicating more severe depressive symptoms. A total score of 0 to 7 generally indicated no depression, 8 to 16 suggested mild depression, 17 to 23 indicated moderate depression, and 24 or above signified severe depression.

The HAMA was specifically designed to assess the severity of anxiety symptoms.^[15]^ It included 14 items covering anxious mood, tension, fear, insomnia, cognitive impairment, depressive mood, somatic anxiety, and autonomic dysfunction. The evaluation was performed by trained professionals through interviews and observations, with each item scored on a scale of 0 to 4. Higher total scores indicated more severe anxiety symptoms. A total score of 0 to 6 generally indicated no anxiety, 7 to 13 suggested mild anxiety, 14 to 20 indicated moderate anxiety, and 21 or above signified severe anxiety.

The clinical efficacy between the 2 groups was compared using a 4-tiered evaluation standard based on symptom improvement and social functional recovery. Markedly effective was defined as the near-complete resolution of core anxiety and depressive symptoms, restoration of social function to pre-illness levels, and a ≥75% reduction in standardized scale scores or scores within the normal range. Effective indicated substantial symptom alleviation with residual mild symptoms, mostly restored social function, and a 50% to 74% reduction in scale scores. Marginally effective referred to partial symptom relief with persistent anxiety or depressive manifestations, impaired social function, and a 25% to 49% reduction in scale scores. Ineffective denoted unchanged or worsened symptoms, severely impaired social function, and a <25% reduction or increased scale scores.

### 2.5. Quality of life

After completing 4 weeks of treatment, the World Health Organization Quality of Life – Brief Version (WHOQOL-BREF) was used to assess the quality of life of patients in both groups.^[16]^ This scale consisted of 26 items covering 4 domains: physical health, psychological health, social relationships, and environment, using a 1-5 Likert scale for scoring. Among them, physical health included 7 items, psychological health 6, social relationships 3, and environment 8. The final scores were calculated separately for each domain, converted proportionally to a percentage system, and the mean values were recorded. Higher scores indicated better quality of life in the respective domain.

### 2.6. Safety assessment

Adverse reactions during the patient’s treatment were recorded, and BMI, fasting blood glucose, and blood lipid levels of both groups were documented before and after treatment. Fasting venous blood was collected from patients, and after centrifugation, fasting blood glucose and blood lipid levels were analyzed using a fully automated biochemical analyzer (BS-200, Mindray).

### 2.7. Statistical analysis

Analyses were performed using IBM SPSS 29.0 statistical software (IBM Corp.). All experimental data conformed to a normal distribution. Categorical data were expressed as frequencies and percentages ([n (%)]) and evaluated using chi-square tests, with the test statistic indicated as χ^2^. Continuous variables were reported as means ± standard deviations and analyzed through independent samples *t* tests, with the test statistic denoted as *t*. Baseline demographic and clinical variables were compared between the 2 groups before treatment to assess comparability, particularly with respect to baseline disease severity. Given the retrospective, non-randomized nature of the study, the statistical comparisons were interpreted as associations rather than proof of causality. A *P* value below .05 was considered indicative of statistical significance.

## 3. Results

### 3.1. Demographic characteristics

The baseline demographic and clinical characteristics of participants in both the olanzapine group (n = 102) and the combined rTMS group (n = 108) were comparable, with no statistically significant differences observed between groups (Table 1). In addition, baseline disease severity was comparable between the 2 groups, as reflected by the absence of significant differences in baseline PSQI and ISI scores (Fig. 1) and baseline HAMD and HAMA scores. These findings indicated that the 2 groups were well-matched in terms of measured baseline demographic variables and measured clinical severity indicators before treatment, although unmeasured confounding could not be excluded because the study was not randomized.

**Table 1:** Comparison of demographic characteristics between 2 groups.

Parameter	Olanzapine group (n = 102)	Combined rTMS group (n = 108)	*t*/χ^2^	*P*
Age (yr)	53.24 ± 8.63	52.85 ± 8.49	0.330	.742
Duration of comorbid Anxiety–depression (wk)	355.98 ± 23.65	352.85 ± 24.12	0.949	.344
Gender				
Female	68 (66.67%)	69 (63.89%)	0.178	.673
Male	34 (33.33%)	39 (36.11%)		
Educational level				
Junior college or below	54 (52.94%)	51 (47.22%)	0.686	.407
Bachelor’s degree or above	48 (47.06%)	57 (52.78%)		
Marital status				
Married	72 (70.59%)	74 (68.52%)	0.106	.745
Unmarried	30 (29.41%)	34 (31.48%)		
Employment status				
Employed	54 (52.94%)	57 (52.78%)	0.001	.981
Retired	48 (47.06%)	51 (47.22%)		
Coffee or tea drinking habit				
Yes	11 (10.78%)	10 (9.26%)	0.136	.713
No	91 (89.22%)	98 (90.74%)		
Other chronic diseases				
Yes	22 (21.57%)	23 (21.3%)	0.002	.962
No	80 (78.43%)	85 (78.7%)		

rTMS = transcranial magnetic stimulation.

**Figure 1.**
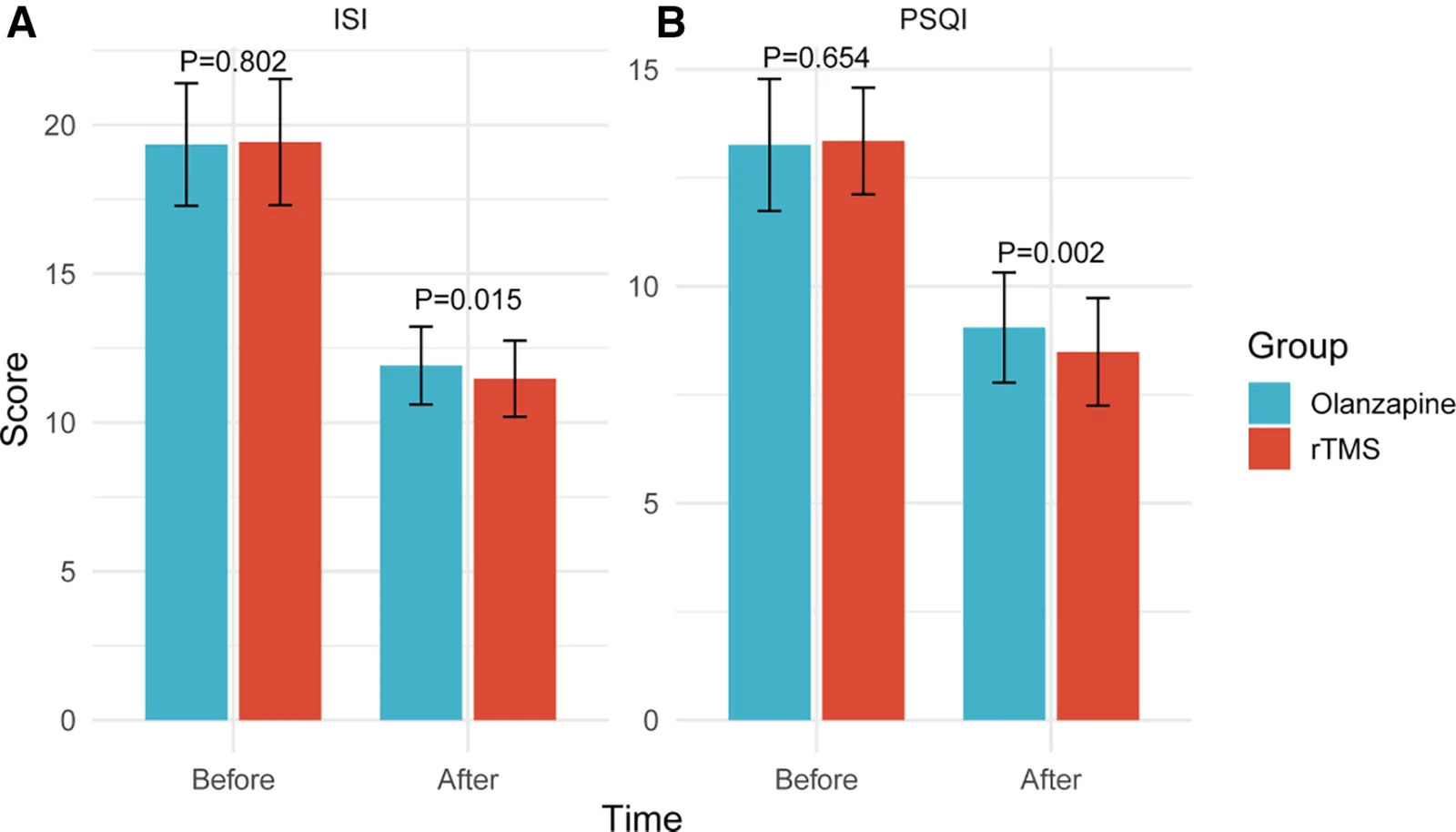
Comparison of sleep quality between the Olanzapine group and the combined rTMS group. (A) Comparison of PSQI; (B) comparison of ISI. ISI = Insomnia Severity Index, PSQI = Pittsburgh Sleep Quality Index, rTMS = transcranial magnetic stimulation.

### 3.2. Sleep-related data

After 4 weeks of treatment, patients in the combined rTMS group demonstrated significantly improved sleep parameters compared with the olanzapine group (Table 2). Sleep efficiency was higher in the combined rTMS group (80.31 ± 9.85%) than in the olanzapine group (76.85 ± 10.53%; t = 2.459, *P* = .015), total sleep time was longer (404.26 ± 14.52 vs 398.26 ± 13.52 minutes; *t* = 3.097, *P* = .002), and the number of nighttime awakenings was reduced (3.78 ± 0.94 vs 4.12 ± 0.85; *t* = 2.747, *P* = .007). These results indicated that the addition of rTMS to olanzapine therapy was associated with greater improvements in sleep quality and continuity in patients with comorbid anxiety–depression and sleep disorders.

**Table 2:** Comparison of sleep-related data between 2 groups.

Parameter	Olanzapine group (n = 102)	Combined rTMS group (n = 108)	*t*	*P*
Efficiency (%)	76.85 ± 10.53	80.31 ± 9.85	2.459	.015
Total time (min)	398.26 ± 13.52	404.26 ± 14.52	3.097	.002
Awakening (no. times)	4.12 ± 0.85	3.78 ± 0.94	2.747	.007

rTMS = transcranial magnetic stimulation.

### 3.3. PSQI and ISI

There were no significant differences in baseline PSQI and ISI scores between the olanzapine group and the combined rTMS group (PSQI: 13.26 ± 1.52 vs 13.35 ± 1.23, *t* = 0.448, *P* = .654; ISI: 19.34 ± 2.06 vs 19.42 ± 2.12, *t* = 0.251, *P* = .802) (Fig. 1). Following 4 weeks of treatment, the combined rTMS group exhibited significantly greater reductions in both PSQI and ISI scores compared with the olanzapine group (PSQI: 8.49 ± 1.24 vs 9.05 ± 1.27, *t* = 3.182, *P* = .002; ISI: 11.47 ± 1.28 vs 11.91 ± 1.31, *t* = 2.448, *P* = .015), indicating superior improvements in sleep quality and insomnia severity among patients receiving combined rTMS and olanzapine treatment.

### 3.4. Symptoms of anxiety and depression and clinical efficacy

There were no significant differences between the olanzapine group and the combined rTMS group in baseline HAMD (21.08 ± 1.45 vs 21.27 ± 1.26, *t* = 1.031, *P* = .304) or HAMA scores (21.89 ± 1.63 vs 22.02 ± 1.68, *t* = 0.540, *P* = .590) (Fig. 2A and B). After 4 weeks of treatment, both groups showed reductions in HAMD and HAMA scores; however, the combined rTMS group achieved significantly greater improvements compared with the olanzapine group (HAMD: 13.51 ± 1.85 vs 14.12 ± 1.49, *t* = 2.627, *P* = .009; HAMA: 12.61 ± 1.78 vs 13.37 ± 1.69, *t* = 3.181, *P* = .002). These findings indicated that the addition of rTMS to olanzapine therapy led to superior improvements in depressive and anxiety symptoms among patients with comorbid anxiety–depression and sleep disorders.

**Figure 2.**
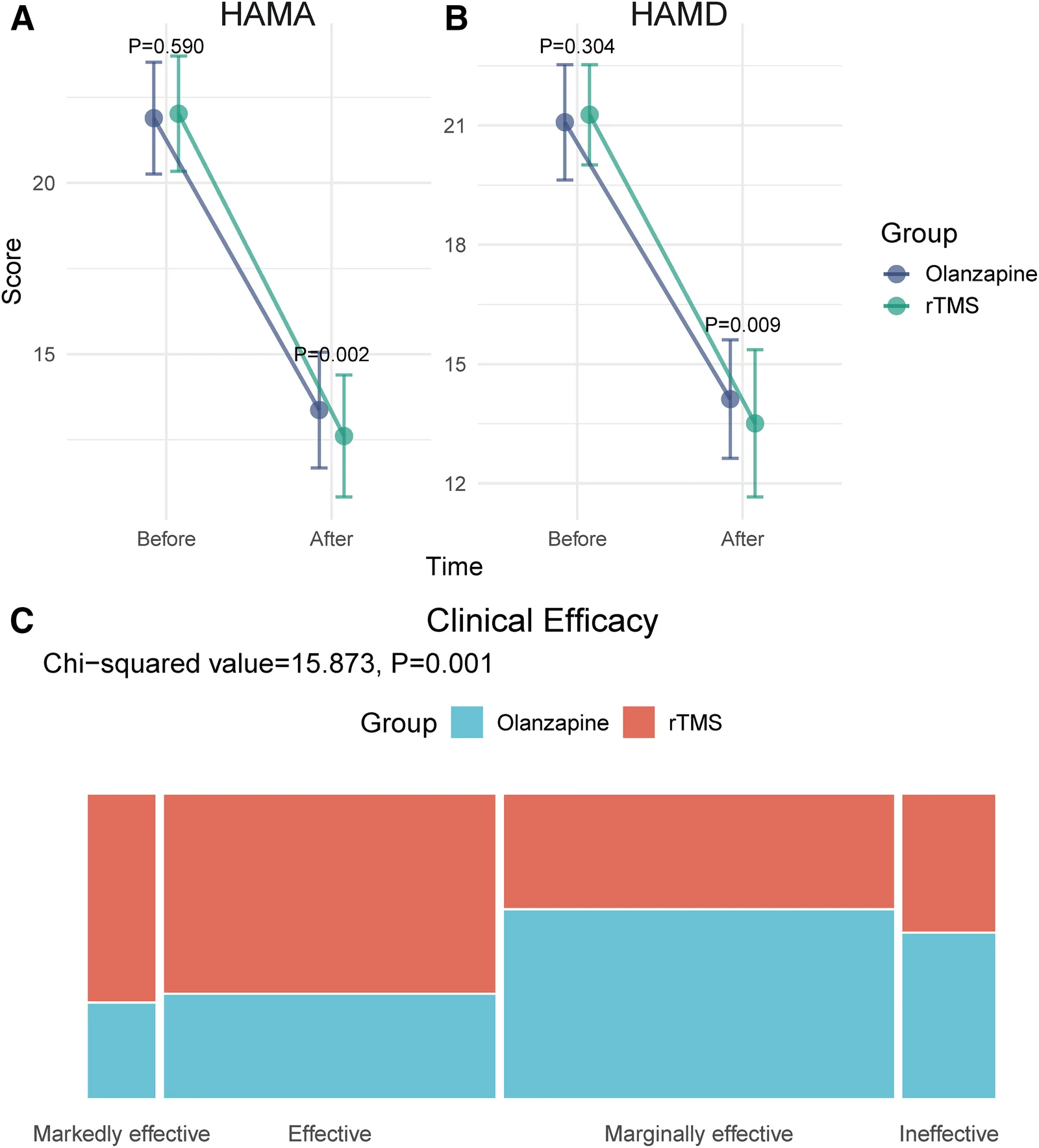
Comparison of HAMD, HAMA, and clinical efficacy between 2 groups. (A) Comparison of HAMD. (B) Comparison of HAMA. (C) Comparison of clinical efficacy. HAMA = Hamilton Anxiety Rating Scale, HAMD = Hamilton Depression Rating Scale, rTMS = transcranial magnetic stimulation.

The combined rTMS group showed significantly greater clinical efficacy compared with the olanzapine group (χ2 = 15.873, *P* = .001) (Fig. 2C). Specifically, the proportion of patients rated as markedly effective was higher in the combined rTMS group (10.19%) than in the olanzapine group (4.90%), and more patients in the combined rTMS group were categorized as effective (48.15% vs 26.47%). Conversely, the proportion of patients considered marginally effective was lower in the combined rTMS group (32.41%) than in the olanzapine group (56.86%), and the rates of ineffective response were similar between groups (9.26% vs 11.76%). These results indicated that olanzapine combined with rTMS achieved superior overall clinical efficacy in patients with comorbid anxiety–depression and sleep disorders.

### 3.5. Quality of life

After 4 weeks of treatment, the combined rTMS group demonstrated significantly higher scores in the WHOQOL-BREF physical health (79.98 ± 2.95 vs 78.65 ± 2.98, *t* = 3.226, *P* = .001) and psychological health domains (76.02 ± 2.75 vs 75.24 ± 2.67, *t* = 2.078, *P* = .039) compared with the olanzapine group (Table 3). There were no significant differences between groups in social relationships (78.16 ± 2.65 vs 78.65 ± 2.98, *t* = 1.253, *P* = .212) or environment domains (85.94 ± 2.28 vs 85.65 ± 2.45, *t* = 0.905, *P* = .367). These findings suggested that the addition of rTMS to olanzapine was associated with improved quality of life, particularly in physical and psychological health domains, among patients with comorbid anxiety–depression and sleep disorders.

**Table 3:** Comparison of WHOQOL-BREF between 2 groups.

Parameter	Olanzapine Group (n = 102)	Combined rTMS Group (n = 108)	*t*	*P*
Physical health	78.65 ± 2.98	79.98 ± 2.95	3.226	.001
Psychological health	75.24 ± 2.67	76.02 ± 2.75	2.078	.039
Social relationships	78.65 ± 2.98	78.16 ± 2.65	1.253	.212
Environment	85.65 ± 2.45	85.94 ± 2.28	0.905	.367

rTMS = transcranial magnetic stimulation, WHOQOL-BREF = World Health Organization Quality of Life – Brief Version.

### 3.6. Side effects

There were no significant differences between the olanzapine group and the combined rTMS group in BMI, fasting blood sugar, cholesterol, or triglyceride levels at baseline or after 4 weeks of treatment. Both groups exhibited comparable changes in BMI (before: 21.64 ± 2.08 vs 21.59 ± 2.16, *t* = 0.192, *P* = .848; after: 23.85 ± 1.74 vs 23.71 ± 1.84, *t* = 0.572, *P* = .568), fasting blood sugar (before: 4.96 ± 0.52 vs 4.92 ± 0.38 mmol/L, *t* = 0.710, *P* = .479; after: 5.46 ± 0.46 vs 5.44 ± 0.42 mmol/L, *t* = 0.332, *P* = .740), cholesterol (before: 176.25 ± 10.62 vs 175.95 ± 11.05 mg/dL, *t* = 0.204, *P* = .839; after: 200.37 ± 14.95 vs 201.05 ± 13.54 mg/dL, *t* = 0.348, *P* = .728), and triglyceride levels (before: 85.62 ± 8.41 vs 85.07 ± 8.45 mg/dL, *t* = 0.477, *P* = .634; after: 110.62 ± 11.72 vs 109.36 ± 10.95 mg/dL, *t* = 0.806, *P* = .421) (Table 4). These findings indicated that the addition of rTMS to olanzapine treatment did not significantly affect metabolic parameters in patients with comorbid anxiety–depression and sleep disorders.

**Table 4:** Comparison of BMI, metabolic parameters, and adverse effects between 2 groups.

Parameter	Olanzapine group (n = 102)	Combined rTMS group (n = 108)	*t*/χ^2^	*P*
BMI				
Before treatment	21.64 ± 2.08	21.59 ± 2.16	0.192	.848
After treatment	23.85 ± 1.74	23.71 ± 1.84	0.572	.568
FBS (mmol/L)				
Before treatment	4.96 ± 0.52	4.92 ± 0.38	0.710	.479
After treatment	5.46 ± 0.46	5.44 ± 0.42	0.332	.740
Cholesterol (mg/dL)				
Before treatment	176.25 ± 10.62	175.95 ± 11.05	0.204	.839
After treatment	200.37 ± 14.95	201.05 ± 13.54	0.348	.728
Triglyceride (mg/dL)				
Before treatment	85.62 ± 8.41	85.07 ± 8.45	0.477	.634
After treatment	110.62 ± 11.72	109.36 ± 10.95	0.806	.421
Adverse effects				
Headache	1 (0.98%)	9 (8.33%)	4.737	.030
Nausea	1 (0.98%)	3 (2.78%)	0.200	.655
Dizziness and fatigue	1 (0.98%)	2 (1.85%)	0	1
Tinnitus or hearing discomfort	0 (0%)	3 (2.78%)	1.240	.265
Neck pain	0 (0%)	1 (0.93%)	None	1
Muscle twitching	0 (0%)	1 (0.93%)	None	1

BMI = body mass index, FBS = fasting blood sugar, rTMS = transcranial magnetic stimulation.

The incidence of most adverse effects was low and similar between the olanzapine group and the combined rTMS group; however, headache was significantly more frequent in the combined rTMS group (8.33%) than in the olanzapine group (0.98%; χ^2^ = 4.737, *P* = .030). There were no significant differences between groups in the rates of nausea (2.78% vs 0.98%; χ^2^ = 0.200, *P* = .655), dizziness and fatigue (1.85% vs 0.98%; χ^2^ = 0, *P* = 1), tinnitus or hearing discomfort (2.78% vs 0%; χ^2^ = 1.240, *P* = .265), neck pain (0.93% vs 0%; *P* = 1), and muscle twitching (0.93% vs 0%; *P* = 1) (Table 4). These findings suggested that, while the addition of rTMS to olanzapine therapy was associated with a higher incidence of headache, other adverse effects were uncommon and did not differ significantly between groups.

## 4. Discussion

The findings of this study provided compelling evidence supporting the efficacy and safety of combining olanzapine with rTMS in the treatment of patients experiencing comorbid anxiety and depression with concurrent sleep disorders. The addition of rTMS to olanzapine therapy led to enhanced improvements in sleep quality, reductions in anxiety and depressive symptoms, and better quality of life compared with olanzapine alone, without aggravating metabolic side effects and with only a mild increase in the rate of headache as an adverse event. This favorable risk–benefit profile indicated potential advantages for an integrative, multimodal approach in managing complex psychiatric comorbidity, particularly where standard pharmacotherapy may have yielded suboptimal results. Several mechanistic explanations underpinned these findings. Olanzapine was a 2nd-generation (atypical) antipsychotic known for its antagonism at various neurotransmitter receptors, including 5-hydroxytryptamine 2A and 2C (serotonin), D2 (dopamine), and H1 (histamine).^[17]^ This broad profile contributed to its efficacy in treating not only psychosis but also depressive and anxiety symptoms, likely by modulating dopaminergic, serotonergic, and noradrenergic pathways involved in mood regulation.^[18]^ Furthermore, olanzapine’s sedative properties, related primarily to its antihistaminic and antiserotonergic actions, rendered it beneficial in alleviating sleep disturbances often comorbid with mood disorders.^[19]^ However, pharmacological interventions alone, particularly with olanzapine, could be associated with slow onset of action, residual symptoms, medication tolerance, and problematic side effects, including metabolic disturbances.^[19]^ This had propelled interest in non-pharmacological adjuncts, with rTMS emerging as a promising tool due to its neuromodulatory effects on cortical excitability and network connectivity.

rTMS involved the focal application of repetitive magnetic pulses to specific brain regions – predominantly the DLPFC, an area implicated in mood regulation, executive function, and sleep-wake processes.^[20,21]^ According to the neuroplasticity hypothesis of depression, the efficacy of rTMS derived from its capacity to restore dysfunctional neural circuits by modulating synaptic plasticity, enhancing neurotrophin expression (notably brain-derived neurotrophic factor), and rebalancing excitatory/inhibitory neurotransmission within prefrontal-limbic networks.^[22]^ Of particular relevance to comorbid anxiety and sleep impairment was the fact that DLPFC-targeted rTMS could exert top–down regulatory control over hyperactive limbic structures (such as the amygdala), which were implicated in both affective dysregulation and perpetuation of insomnia.^[23]^ Chronic insomnia and sleep disruption perpetuated a state of heightened arousal and anxiogenic neurocircuitry, further fueling mood disturbances.^[24]^ The capacity of rTMS to suppress cortical hyperarousal, modulate autonomic tone, and normalize sleep architecture – through mechanisms such as re-entrainment of thalamo-cortical oscillations – likely contributed to the observed improvements in sleep efficiency, duration, and continuity.^[25]^

Moreover, accumulating evidence suggested a bidirectional relationship between sleep and emotional regulation.^[26,27]^ Sleep disturbances precipitated or exacerbated mood disorders, while effective treatment of insomnia accelerated and potentiated antidepressant and anxiolytic responses.^[28]^ The significant improvements in PSQI and ISI scores in the combined rTMS group, therefore, reflected direct modulation of sleep-related brain networks but could also serve as an important mediator of overall symptomatic and functional recovery in this cohort. It was noteworthy that while both groups manifested improvements in mood and sleep parameters, the magnitude of reduction in standardized scales (HAMD, HAMA, PSQI, and ISI) was consistently greater in the group receiving combined therapy. This observation aligned with the current understanding that circuit-based interventions could enhance and accelerate the effects of pharmacological treatment by directly influencing neural activity patterns that remained resistant to drug modulation alone.^[29,30]^ The faster and more robust improvements in clinical symptoms likely stemmed from synergistic action – olanzapine modulating neurotransmitter output and rTMS directly rectifying aberrant regional activity and promoting neuroplasticity.^[31]^

Quality of life, as measured by the WHOQOL-BREF, was also more substantially improved in the physical and psychological domains in the combined rTMS group, highlighting the functional relevance of neurobiological restoration. Improvements in physical health resulted from restorative sleep, increased daytime vigor, and amelioration of psychomotor symptoms; psychological health benefits, in turn, arose from relief of anxious and depressive affect, better cognitive clarity, and improved resilience, all mediated by restoration of healthy brain rhythms and improved synaptic efficiency.

Importantly, the safety profile of the combined regimen deserved emphasis. Olanzapine was well-documented to increase risks of metabolic dysregulation, including weight gain, hyperglycemia, and dyslipidemia, limiting its use in vulnerable populations.^[32,33]^ Concerns had been raised that additional interventions might compound these risks.^[34]^ However, in this study, metabolic indices (BMI, blood glucose, and lipid profiles) remained comparable between the 2 groups at baseline and study end, suggesting that rTMS did not exacerbate and might conceivably mitigate metabolic side effects through indirect modulation of hypothalamic–pituitary–adrenal axis dynamics or improved sleep and activity patterns. The only adverse effect observed with increased frequency in the rTMS group was headache, a well-known transient side effect likely related to stimulation of superficial neural and muscular structures; this was infrequent and mild, consistent with prior safety data for rTMS.^[35]^

These findings supported the integration of rTMS as an adjunctive therapy in patients who had not responded adequately to pharmacotherapy or who presented with complex symptom constellations such as co-occurring mood and sleep disorders. The multimodal approach allowed for lower drug dosages, potentially lessening dose-dependent side effects. The observed improvements in clinical efficacy, sleep regulation, and quality of life highlighted the importance of considering both neurochemical and neurocircuitry targets in treatment paradigms. From a translational perspective, the potential for rTMS to simultaneously address both affective and sleep-wake pathophysiology echoed the converging neuroscientific understanding of depression, anxiety, and insomnia as disorders of interacting brain networks, not isolated neurochemical imbalances.^[36,37]^

This study also aligned with burgeoning research38 advocating for objective monitoring of sleep and behavior in neuropsychiatric trials – here, the use of actigraphy enabled quantification of sleep quality beyond self-report, lending greater robustness to outcome measurement.^[38]^ Such methodological refinements strengthened the validity of treatment comparisons and facilitated more nuanced approaches to personalized medicine. Nevertheless, the present findings should be interpreted cautiously because of several important methodological limitations. First, this was a retrospective, non-randomized cohort study, and group assignment was based on the treatment actually received in routine clinical practice rather than random allocation. Although both groups met the same diagnostic and eligibility criteria, and baseline demographic characteristics as well as baseline HAMD, HAMA, PSQI, and ISI scores were statistically comparable, the decision to add rTMS may have been influenced by physician judgment, patient preference, treatment accessibility, perceived need for adjunctive intervention, or tolerance considerations. These factors may introduce selection bias and may also limit causal inference. Second, potential confounding variables could not be completely controlled. We compared measured baseline variables between groups; however, unmeasured factors such as prior treatment history, psychotherapy exposure, medication adherence, socioeconomic status, lifestyle factors, illness chronicity, and patient expectations may have affected treatment response. Therefore, the observed superiority of combined therapy should be interpreted as an association rather than definitive evidence of causality. Third, the follow-up period was limited to 4 weeks, which was sufficient to evaluate short-term symptom changes but was too short to determine the persistence of therapeutic benefit, relapse rates, delayed adverse events, or long-term metabolic outcomes associated with olanzapine. The sample was also limited to a single hospital, which may restrict generalizability to broader patient populations. Future prospective, multicenter, randomized controlled trials with longer follow-up, standardized treatment indications, and adjustment for potential confounders are needed to confirm these findings and to identify patients most likely to benefit from adjunctive rTMS.

## 5. Conclusions

In summary, the present study suggested that augmenting olanzapine with rTMS may provide short-term symptomatic relief, functional improvement, and acceptable tolerability in individuals with comorbid anxiety–depression and sleep dysfunction. The results illustrated the importance of addressing both neurochemical imbalance and neural circuit dysfunction in complex psychiatric presentations. However, because this study was retrospective, non-randomized, and limited to a 4-week follow-up period, the findings should be regarded as preliminary and hypothesis-generating. Prospective randomized studies with longer follow-up are warranted to verify the durability, safety, and generalizability of this combined treatment strategy.

## Author contributions

**Conceptualization:** Deyang Yin, Da Yang, Xuan Hou, Ying Liu.

**Data curation:** Deyang Yin, Da Yang, Xuan Hou, Ying Liu.

**Formal analysis:** Deyang Yin, Da Yang, Xuan Hou, Ying Liu.

**Funding acquisition:** Deyang Yin.

**Investigation:** Deyang Yin.

**Writing – original draft:** Deyang Yin.

**Writing – review & editing:** Deyang Yin.
